# *Dmrt1* polymorphism covaries with sex‐determination patterns in *Rana temporaria*

**DOI:** 10.1002/ece3.2209

**Published:** 2016-05-30

**Authors:** Wen‐Juan Ma, Nicolas Rodrigues, Roberto Sermier, Alan Brelsford, Nicolas Perrin

**Affiliations:** ^1^ Department of Ecology and EvolutionUniversity of LausanneCH 1015LausanneSwitzerland; ^2^ Present address: Department of BiologyUniversity of California at RiversideCalifornia92521

**Keywords:** Gonadal development, homomorphic sex chromosomes, nonrecombining segment, sex determination, sex races, sex reversal

## Abstract

Patterns of sex‐chromosome differentiation and gonadal development have been shown to vary among populations of *Rana temporaria* along a latitudinal transect in Sweden. Frogs from the northern‐boreal population of Ammarnäs displayed well‐differentiated X and Y haplotypes, early gonadal differentiation, and a perfect match between phenotypic and genotypic sex. In contrast, no differentiated Y haplotypes could be detected in the southern population of Tvedöra, where juveniles furthermore showed delayed gonadal differentiation. Here, we show that *Dmrt1*, a gene that plays a key role in sex determination and sexual development across all metazoans, displays significant sex differentiation in Tvedöra, with a Y‐specific haplotype distinct from Ammarnäs. The differential segment is not only much shorter in Tvedöra than in Ammarnäs, it is also less differentiated and associates with both delayed gonadal differentiation and imperfect match between phenotypic and genotypic sex. Whereas Tvedöra juveniles with a local Y haplotype tend to ultimately develop as males, those without it may nevertheless become functional XX males, but with strongly female‐biased progeny. Our findings suggest that the variance in patterns of sex determination documented in common frogs might result from a genetic polymorphism within a small genomic region that contains *Dmrt1*. They also substantiate the view that recurrent convergences of sex determination toward a limited set of chromosome pairs may result from the co‐option of small genomic regions that harbor key genes from the sex‐determination pathway.

## Introduction

In sharp contrast to the highly differentiated W and Y chromosomes found in most birds and mammals, sex chromosomes are often homomorphic in cold‐blooded vertebrates (Schmid and Steinlein 2001; Devlin and Nagahama 2002; Schmid et al. 2010). Homomorphy may result from occasional XY recombination (Stöck et al. 2011; Guerrero et al. 2012) and/or high rates of sex‐chromosome turnover (Hillis and Green 1990; Schartl 2004; Volff et al. 2007; Evans et al. 2012), two mechanisms possibly stemming from incomplete genetic control over sex determination (Perrin 2009; Grossen et al. 2011). Both XY recombination and sex‐chromosome turnovers have been documented in amphibians (e.g., Stöck et al. 2013; Dufresnes et al. 2015), where approximately 96% of species lack morphologically differentiated sex chromosomes (Schmid et al. 1991; Eggert 2004).

**Figure 1:**
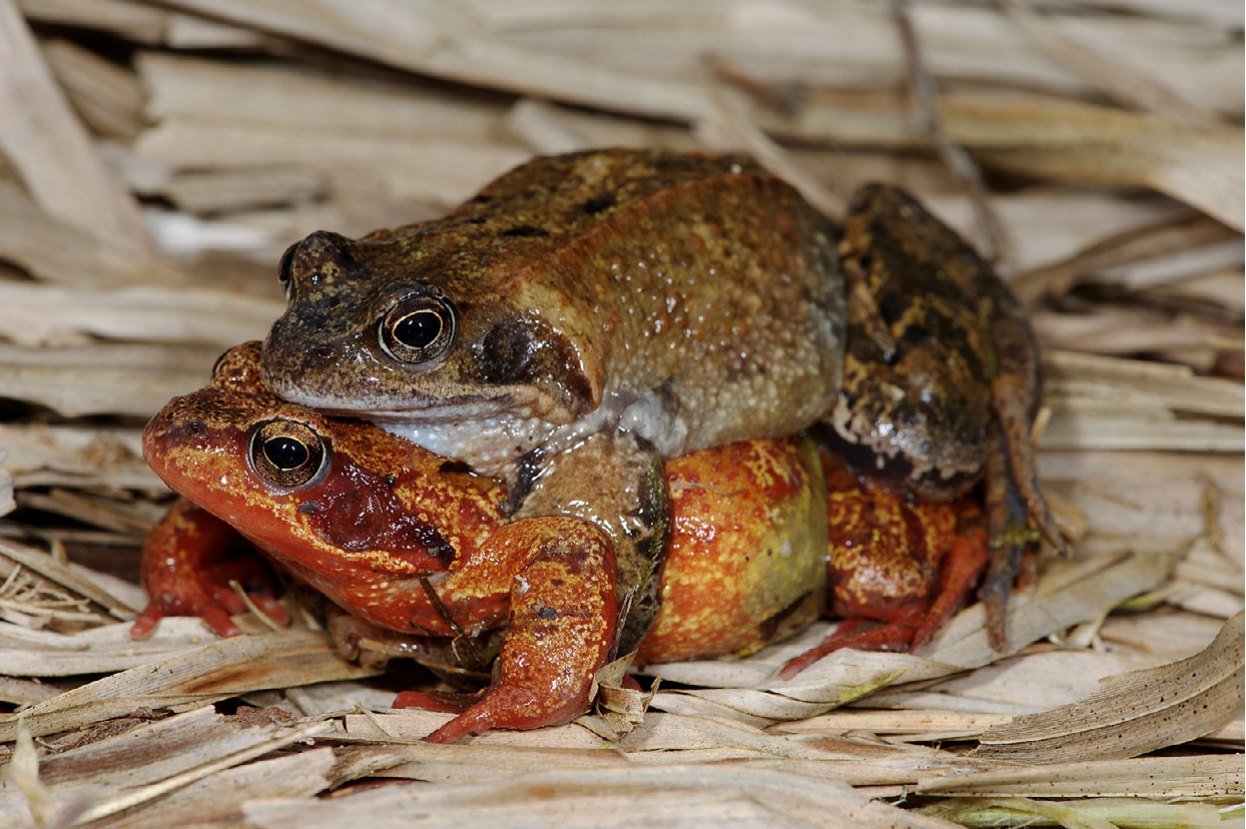
Mating pair of *Rana temporaria* in amplexus. Photography credit Andreas Meyer.

Such is the case of the common frog, *Rana temporaria* (Fig. 1), a European species widely distributed from Spain to northern Norway. Sex determination in common frogs associates with linkage group 2 (LG_2_), as initially indicated by sex differences in allele frequencies at a series of microsatellite markers (Matsuba et al. 2008; Alho et al. 2010; Cano et al. 2011). However, genetic differentiation between sex chromosomes was shown to vary among populations along a latitudinal transect across Fennoscandia (Rodrigues et al. 2014). In the northern‐boreal population of Ammarnäs, all males had fixed specific alleles at LG_2_ markers, forming distinct X and Y haplotypes. In contrast, the same markers failed to identify any sex differentiation in the southern population of Tvedöra: individuals of both sexes harbored the same alleles at similar frequencies, testifying to regular recombination. Intermediate populations displayed a mixed situation: some males had distinct Y haplotypes, while others were genetically indistinguishable from females.

Family analyses revealed that the contrast between Ammarnäs and Tvedöra did not stem from differences in sex‐specific patterns of recombination, but in the mechanisms of sex determination (Rodrigues et al. 2015). Juveniles from Ammarnäs families displayed balanced sex ratios already at metamorphosis (a feature characterizing the “differentiated” sex race; Witschi 1929, 1930), and strong associations between phenotypic sex and paternally inherited LG_2_ haplotypes. In Tvedöra, by contrast, a majority of offspring presented ovaries at metamorphosis (a feature of the “semidifferentiated” sex race); sex ratios were more balanced at the froglet stage, but still variable among families, being male‐biased in some and female‐biased in others. Associations between offspring sex and paternal LG_2_ haplotype were much weaker than in Ammarnäs, and variable among families, but still highly significant overall, a surprising result given the absence of male‐specific alleles at all LG_2_ markers investigated. Genotyping of markers from other linkage groups failed to find any sex association outside LG_2_ in Tvedöra (Rodrigues et al. 2016).

Altogether, these results show that LG_2_ contributes to sex determination in both populations, but in different ways. In Ammarnäs, alleles at the sex locus associate with early gonadal differentiation (the “differentiated race” syndrome) and strictly genetic sex determination (GSD). Because XY individuals always develop as males (which only recombine in the distal parts of chromosomes; Brelsford et al. 2016a, 2016c), recombination is arrested over most of the sex chromosome, resulting in marked XY differentiation. In Tvedöra, by contrast, alleles at the sex locus associate with delayed gonadal differentiation (the “semidifferentiated race” syndrome) and imperfect match between genetic and phenotypic sex (“leaky GSD”). Occasional events of sex reversal might account for the variance in sex ratios among families (excess of sons in the progeny of XY females, excess of daughters in the progeny of XX males), as well as for the absence of sex‐chromosome differentiation (resulting from XY recombination in XY females – the fountain‐of‐youth model; Perrin 2009; Matsuba et al. 2010).

Importantly (and independent of the underlying mechanisms), the situation in Tvedöra offers a unique opportunity to search for the sex locus. Contrasting with Ammarnäs, where sex chromosomes are differentiated over most of their length, occasional recombination in Tvedöra is expected to regularly restore XY similarity all along the chromosome, except for the immediate neighborhood of the sex‐determining locus. This should greatly facilitate its identification, by narrowing its localization down to a restricted nonrecombining sex‐determining region (SDR) displaying significant XY differentiation.

This study focuses on *Dmrt1*, an important gene from the sex‐determining cascade mapping to LG_2_ in *R. temporaria* (Brelsford et al. 2013). This gene or paralogs participate in sex determination and/or sexual dimorphism throughout the animal kingdom (Beukeboom and Perrin 2014); it plays a central sex‐determining role in birds (Smith et al. 2009), while paralogs take this role in several fish and frogs (Matsuda et al. 2002; Nanda et al. 2002; Yoshimoto et al. 2008). It thus qualifies as a potential candidate sex‐determining gene in our focal species. We identified three polymorphic markers in distinct noncoding parts of *Dmrt1* and two more in the genes immediately flanking *Dmrt1* in the *X. tropicalis* genome (namely *Kank1* upstream and *Dmrt3* downstream) and analyzed them for sex association in adults and families from Ammarnäs and Tvedöra. Our first aim was to test whether these markers showed any sex differentiation in Tvedöra, which would indicate proximity to the sex locus, given the occasional recombination and absence of sex differentiation for all other LG_2_ markers analyzed so far. In case of a positive result, our second aim was to investigate whether polymorphism at these markers might correlate with the variation in sex‐determination patterns documented among Tvedöra families (Rodrigues et al. 2015), in particular regarding the suggested occurrence of sex‐reversed XX males and XY females.

## Materials and Methods

### Field sampling and husbandry

The same samples were used as in Rodrigues et al. (2015). Mating pairs were caught in amplexus during the 2013 breeding season from two Swedish populations: 20 pairs from the northern‐boreal population of Ammarnäs (65°58′12.60″N, 16°12′43.80″E) and 11 pairs from the southern population of Tvedöra (55°42′0.85″N, 13°25′50.91″E). Buccal cells were sampled with sterile cotton swabs before release at the place of capture. Clutches of six pairs from each population (SA1‐SA6 and ST1‐ST6) were reared in outdoor facilities on the campus of the University of Lausanne. Within 1 week of metamorphosis, 40 offspring from each clutch (referred to as “metamorphs”) were anaesthetized and euthanized in 0.2% ethyl3‐aminobenzoate methanesulfonate salt solution (MS222), then dropped in 70% ethanol for preservation at −20°C. The remaining offspring (referred to as “froglets”) were allowed to grow for a few more weeks and similarly euthanized when reaching about 2 cm snout–vent length (Gosner stage 46; Gosner 1960).

### Progeny sexing

Metamorphs and froglets were dissected under a binocular microscope in order to determine the phenotypic sex based on gonad morphology. These stages were chosen because “sex races” are defined by their differences in the patterns of gonadal development at metamorphosis (Witschi 1929): contrasting with the “differentiated sex race,” where juveniles present already at metamorphosis testes or ovaries in equal proportions, juveniles from the “semidifferentiated race” mostly present ovaries at this stage (so that discrepancies are expected between genetic and phenotypic sex). Only later in development (at the froglet stage and later) do some of these juveniles replace ovaries by testes (Witschi 1929). Ovaries in common frogs develop from the whole gonadal primordia into a large whitish/yellowish structure with distinct lobes and a characteristic granular aspect conferred by the many oocytes embedded in the cortex (Ogielska and Kotusz 2004). In contrast, testes develop from the anterior part of the gonadal primordia only (the posterior part degenerates) into a small oblong structure, with a smooth cortex covered with melanic spots (Haczkiewicz and Ogielska 2013). As gonads are not always well differentiated externally at metamorphosis, we applied a semiquantitative scale to score individuals along a gradient of apparent maleness. Individuals with distinctive male or female gonads were assigned scores of 1.0 and 0.0, respectively. Individuals identified as “likely” males or females were assigned scores of 0.9 and 0.1, respectively, while others identified as “possibly” males or females were scored as 0.7 and 0.3, respectively. Individuals with undifferentiated gonads were scored as 0.5. Note that only relative score values matter here, because we applied rank statistics (see “Statistical analyses”). All individuals were scored independently by N. Rodrigues and Y. Vuille before genetic analyses (summer 2013), with concordant results (correlation > 0.95).

### Marker development

After overnight treatment with 10% proteinase K (Qiagen) at 56°C, DNA was extracted from hindleg tissues (metamorphs and froglets) and buccal swabs (adults) using a Qiagen DNeasy kit and a BioSprint 96 workstation (Qiagen), resulting in a 200 *μ*L Buffer AE (Qiagen) DNA elution.

The cDNA *Dmrt1* sequence of *Rana chensinensis* was downloaded from NCBI gene database. Blasts against the *R. temporaria* low‐coverage draft genome (Brelsford et al. 2016c) returned five scaffolds as the best hits, each including a full or partial *Dmrt1* exon (Appendix S1, Text S1). Exon–intron boundaries were identified by comparing genomic DNA (gDNA) sequences to the cDNA sequences obtained from five froglets (Appendix S1, Text S2). RNA extraction was performed following the standard Trizol protocol. In short, snap frozen froglet samples were individually homogenized in Trizol (Life Technologies), followed by phase separation (using chloroform); after ethanol precipitation of the upper phase, RNA was washed with 70% ethanol twice and collected. cDNA was synthesized using Superscript III Reverse Transcriptase (Life Technologies), after DNAse treatment which removed any gDNA contamination.

**Figure 2:**
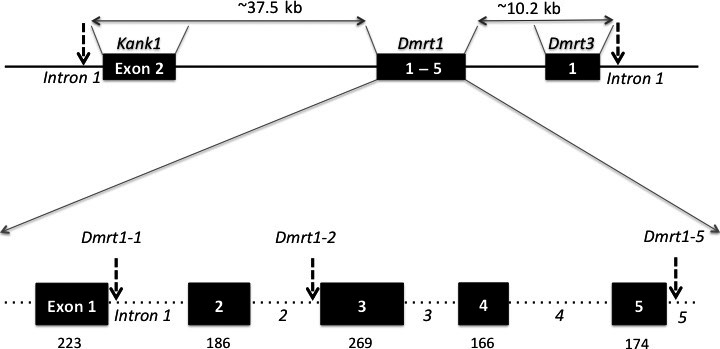
Structure of the genomic region investigated here, with localization of the five length‐polymorphic markers analyzed (arrows). Top: In *X. tropicalis*,* Kank1* is the closest gene upstream of *Dmrt1*, and *Dmrt3* the closest downstream. The distances indicated correspond to *X. tropicalis*, and might be longer in *R. temporaria*, because of its larger genome. Bottom: enlargement of *Dmrt1*; boxes denote the five exons with their respective sizes (in bp) indicated underneath. Dotted lines between exons represent introns of unknown size in *R. temporaria*.

Primer pairs (Appendix S2, Table S1) were designed in the intron regions flanking exons (<200 bp each direction); for exons 2 and 5, one flanking region (3′ and 5′, respectively) was missing from the scaffolds, so that the corresponding primers were designed within exons. With these primers, we amplified and sequenced (Microsynth) fragments from 26 individuals (14 from Ammarnäs and 12 from Tvedöra). Ambiguous fragment sequences were cloned before sequencing, using TOPO^®^ TA Cloning^®^ Dual Promoter Kit with One Shot^®^ TOP10 chemically competent *E. coli* cells, following the protocol provided by the manufacturer. Besides multiple synonymous SNPs within exons, three length‐polymorphic sites were detected in different noncoding regions (Appendix S1, Text S3), corresponding to a microsatellite repeat in the 5′ part of intron 1, an indel in the 3′ part of intron 2, and a single nucleotide repeat (cytosine) in the 3’ UTR region of exon 5 (Fig. 2). Specific fluorescent primers (Appendix S2, Table S2) were designed for all three length‐polymorphic sites.

As we did not aim at characterizing X‐ and Y‐sequences for *Kank1* and *Dmrt3* (because they do not qualify as candidate sex‐determining genes), we used a simpler procedure to develop length‐polymorphic markers. All scaffolds of the *R. temporaria* low‐coverage draft genome (Brelsford et al. 2016c) were aligned to the *X. tropicalis* genome with Blastn. *Rana* scaffolds mapping to *X. tropicalis* genes *Kank1* and *Dmrt3* (Appendix S1, Text S1) were screened for microsatellite markers using the microsatellite identification tool MISA (http://pgrc.ipk-gatersleben.de/misa/), and specific fluorescent primers were designed in the flanking regions of the microsatellite with longest repeat motif for each gene (both are on intron 1, Fig. 2; Appendix S2, Table S2).

### Genotyping

All adults and juveniles from Ammarnäs and Tvedöra were then genotyped for these five length‐polymorphic markers. PCRs were performed in a total volume of 10 *μ*L, including 3 *μ*L of extracted DNA, 2.22 *μ*L of Milli‐Q water, 3 *μ*L of Qiagen Multiplex Master Mix, and 0.14–0.3 *μ*L of labeled forward primer and 0.14–0.3 *μ*L of unlabeled reverse primer (in total 1.78 *μ*L of primer mix). PCRs were conducted on Perkin Elmer 2700 machines using the following thermal profile: 15 min of Taq polymerase activation at 95°C, followed by 35 cycles including denaturation at 94°C for 30 sec, annealing at 55°C for 1.5 min and elongation at 72°C for 1 min, ending the PCR with a final elongation of 30 min at 60°C. PCR products for genotyping were run on an automated ABI Prism 3100 sequencer (Applied Biosystems, Foster City, CA), and alleles were scored using GENEMAPPER v. 4.0 (Applied Biosystems).

### Statistical analyses

Associations between offspring sex‐phenotype scores and paternally inherited LG_2_ haplotypes were quantified with Somers’ (1962) *Dxy* rank correlation (a measure of association between an ordinal variable *x* and a binary variable *y*) and tested with nonparametric Wilcoxon–Mann–Whitney (WMW) tests (statistics performed in R, v3.1.1, R Core Team, 2014). Between‐sex *F*_ST_ values were calculated and tested (10,000 permutations) among adults from Ammarnäs and Tvedöra (FSTAT v2.9.3, updated from Goudet 1995). *F*_ST_ values for the five markers were compared to those obtained for the 13 LG_2_ markers genotyped on the same sample by Rodrigues et al. (2015). Family genotypes were also combined with those obtained at these 13 LG_2_ markers, in order to localize our five markers on the consensus recombination map. Sex‐specific recombination rates were estimated with CRIMAP v2.4 (Green et al. 1990). The *twopoint* option was used to identify marker pairs with a LOD score exceeding 3.0, the *all* option to generate loci order, the *build* option to calculate the distances between loci (centimorgans, cM), and the *flip* option to test the robustness of loci order. A female consensus recombination map was plotted using MAPCHART v2.2 (Voorrips 2002).

## Results

In adults from Ammarnäs, all five markers displayed sex‐diagnostic differences in allele frequencies (Table 1). All 20 males possessed at each locus exactly one copy of a male‐specific allele, not found in any female. As a result, *F*_ST_ between sexes were high and significant for all five loci (average 0.286, range 0.142–0.514, all *P* values ~0.0002 after correction for multiple testing; Appendix S2, Table S3). Sibship analyses confirmed that alleles identified as male specific were indeed located on nonrecombining Y haplotypes. The most common haplotype had fixed allele *171* at *Kank1*,* 337* at *Dmrt1‐1*,* 212* at *Dmrt1‐2*,* 296* at *Dmrt1‐5*, and *291* at *Dmrt3*. Two other closely related Y haplotypes were found, differing at one or two loci (changes to allele *335* at *Dmrt1‐1* and/or *285* at *Dmrt3*). These analyses also revealed a highly significant association between inheritance of male‐specific Y haplotypes and offspring phenotypic sex, both in metamorphs (*n* = 240, Somer's *Dxy* rank correlation* *= 0.71, *P *<* *2.2 × 10^−16^, WMW test) and in froglets (*n* = 31, *Dxy* = 1.0, *P *=* *4.9 × 10^−8^, WMW test; Table 2). Correlations were also significant in all families separately (*n* = 41–49 in each, *Dxy* varying from 0.60 to 0.95, all *P *< 10^−6^), except for family SA_6_ where gonads were still undifferentiated in metamorphs.

**Table 1:** Sex‐specific allele frequencies in Ammarnäs (*n* = 40) and Tvedöra (*n* = 22)

Marker	Allele size	Ammarnäs		Tvedöra	
		Female	Male	Female	Male
*Kank1*	165	0.00	0.00	0.23	0.14
	168	0.00	0.00	0.00	0.05
	171	0.00	**0.50**	0.00	0.00
	174	1.00	0.50	0.77	0.73
	178	0.00	0.00	0.00	0.09
*Dmrt1‐1*	291	0.73	0.43	0.09	0.14
	292	0.28	0.08	0.64	0.41
	294	0.00	0.00	0.00	**0.41**
	325	0.00	0.00	0.27	0.05
	335	0.00	**0.05**	0.00	0.00
	337	0.00	**0.45**	0.00	0.00
*Dmrt1‐2*	198	0.30	0.08	0.95	0.86
	211	0.70	0.42	0.05	0.14
	212	0.00	**0.50**	0.00	0.00
*Dmrt1‐5*	296	0.00	**0.50**	0.23	0.09
	300	0.00	0.00	0.18	0.14
	301	0.08	0.00	0.55	0.64
	302	0.20	0.11	0.05	0.05
	303	0.00	0.05	0.00	0.05
	304	0.73	0.34	0.00	0.00
	305	0.00	0.00	0.00	0.05
*Dmrt3*	276	0.13	0.03	0.59	0.45
	281	0.00	0.00	0.00	**0.36**
	285	0.00	**0.16**	0.00	0.00
	287	0.05	0.00	0.00	0.00
	290	0.03	0.00	0.00	0.00
	291	0.00	**0.34**	0.09	0.05
	293	0.00	0.03	0.00	0.00
	297	0.00	0.00	0.23	0.09
	300	0.66	0.37	0.09	0.05
	303	0.00	0.03	0.00	0.00
	309	0.13	0.05	0.00	0.00

Male‐specific alleles are indicated in bold.

**Table 2:** Numbers of metamorphs and froglets per maleness score (from 0 to 1) as a function of presence (+) or absence (−) of a Y‐specific *Dmrt1‐1* allele (*294* in Tvedöra, *335* or *337* in Ammarnäs) in six families from Ammarnäs (SA1 to SA6) and Tvedöra (ST1 to ST6). Also provided are Somers rank correlation values (*Dxy*), sample sizes (*n*), and *P* values from Wilcoxon–Mann–Whitney tests (*P*)

Family		Metamorphs									Froglets											Total		
		0	0.1	0.3	0.5	0.7	0.9	1	*Dxy*	*n*	*P*	0	0.1	0.3	0.5	0.7	0.9	1	*Dxy*	*n*	*P*	*Dxy*	*n*	*P*
SA1	−	21	0	0	0	0	0	0	0.75	40	1.2e‐08***	7	0	0	0	0	0	0	1	9	0.009*	0.8	49	1.2e‐10***
	+	1	0	0	6	0	0	12				0	0	0	0	0	0	2						
SA2	−	22	0	0	0	0	0	0	0.58	40	1.9e‐09***	2	0	0	0	0	0	0	1	3	0.48	0.6	43	4.7e‐10***
	+	0	0	0	13	3	0	2				0	0	0	0	0	0	1						
SA3	−	16	0	1	9	0	0	0	0.93	40	2.1e‐07***	2	0	0	0	0	0	0	1	7	0.03*	0.93	47	3.3e‐09***
	+	0	0	0	2	0	0	12				0	0	0	0	0	0	5						
SA4	−	22	0	0	0	0	0	0	0.92	40	9.4e‐10***	1	0	0	0	0	0	0	NA	1	NA	0.92	41	5.5e‐10***
	+	0	0	0	1	1	0	16				0	0	0	0	0	0	0						
SA5	−	17	0	0	1	0	0	0	0.95	40	1.4e‐09***	3	0	0	0	0	0	0	1	7	0.046*	0.95	47	6.4e‐11***
	+	0	1	0	1	0	0	20				0	0	0	0	0	0	4						
SA6	−	0	0	0	18	0	0	0	NA	40	NA	4	0	0	0	0	0	0	1	4	0.19	NA	44	0.04*
	+	0	0	0	22	0	0	0				0	0	0	0	0	0	0						
Ammarnäs	−	98	0	1	28	0	0	0	0.71	240	<2.2e‐16***	19	0	0	0	0	0	0	1	31	4.9e‐08***	0.74	271	<2.2e‐16***
	+	1	1	0	45	4	0	62				0	0	0	0	0	0	12						
ST1	−	40	0	0	0	0	0	0	NA	40	NA	10	0	0	0	0	0	1	0	11	NA	0	51	NA
	+	0	0	0	0	0	0	0				0	0	0	0	0	0	0						
ST2	−	4	0	0	20	0	0	0	0.62	40	0.046*	0	0	0	0	0	0	4	NA	7	NA	0.12	47	0.18
	+	0	0	0	15	0	0	1				0	0	0	0	0	0	3						
ST3	−	22	0	0	0	0	0	0	0.58	40	3.0e‐05***	2	0	0	0	0	0	1	0.58	15	0.04*	0.51	55	1.2e‐06***
	+	7	2	5	0	1	1	2				1	0	0	0	0	0	11						
ST4	−	23	1	0	0	0	0	1	0.42	40	0.04*	4	0	0	0	0	0	2	0.13	22	0.22	0.37	62	0.0007**
	+	10	0	1	1	0	0	3				4	0	0	4	0	0	8						
ST5	−	16	0	0	0	0	0	0	0.52	40	2.8e‐05***	8	0	0	0	0	0	0	0.89	20	3.0e‐05***	0.59	60	2.7e‐08***
	+	7	3	3	2	0	0	9				0	0	0	1	0	0	11						
ST6	−	13	0	1	0	0	0	0	0.39	40	0.0006**	2	0	0	1	0	0	0	1	8	0.016*	0.44	48	0.0001***
	+	9	3	1	7	0	2	4				0	0	0	0	0	0	5						
Tvedöra	−	117	1	1	20	0	0	1	0.59	240	3.8e‐15***	26	0	0	1	0	0	8	0.56	83	2.2e‐08***	0.51	323	2.2e‐16***
	+	34	8	10	25	1	3	19				5	0	0	5	0	0	38						

NA, not applicable. *, *P* < 0.05; **, *P* < 0.001; ***, *P* < 0.0001.

**Figure 3:**
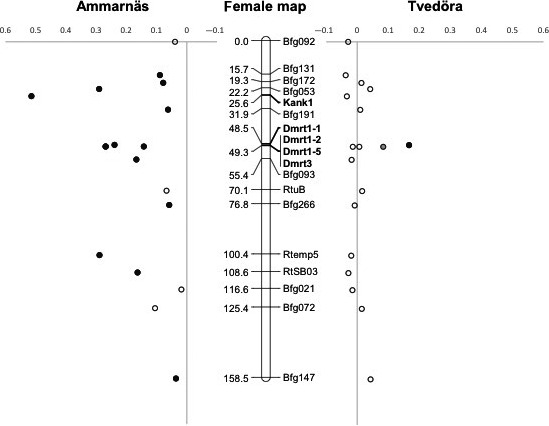
Consensus female recombination map based on all 12 families from Ammarnäs and Tvedöra. Between‐sex *F*_ST_ values are indicated for each marker, either left (Ammarnäs) or right (Tvedöra). Indicated in bold are the five markers developed here. Loci with significant *F*_ST_ values are indicated by black symbols, and *Dmrt3* in Tvedöra (with a distribution of the male‐specific allele that departs significantly from random) by a gray symbol.

In Tvedöra, male‐specific alleles were found at *Dmrt1‐1* and *Dmrt3* (alleles *294* and *281,* respectively, Table 1), both of which were however missing in two males of 11. *F*_ST_ values for these markers reached 0.167 and 0.084, respectively (with *P* values < 0.004 and 0.087 after correction for multiple testing, Appendix S2, Table S3). Although the *F*_ST_ value associated with *Dmrt3* is only close to significance after correction, the exact probability for the observed distribution of the male‐specific allele can be computed from combinatorial statistics as the ratio of 2^8^ × 11!/(8! × 3!) = 42,240 (number of combinations of eight copies of allele *281* among 11 males, one copy each) over 44!/(8! × 36!) = 177,232,627 (number of combinations of these eight copies among 44 copies of *Dmrt3*), which amounts to *P* ~ 2.4 × 10^−4^. If we furthermore account for the fact that these copies only occurred in males that otherwise possess allele *294* at *Dmrt1‐1*, the probability becomes *P* ~ 1.3 × 10^−5^. The three other loci did not show significant sex differences in allele frequencies. Between‐sex *F*_ST_ values averaged 0.042 over the five markers (as compared to −0.0005 over all other LG_2_ markers; Rodrigues et al. 2015). Locus‐specific *F*_ST_ values are plotted along the consensus female recombination map in Figure 3, showing the contrasted patterns of sex differentiation between populations, and localizing the small differential segment in Tvedöra, identified through *Dmrt1‐1* and *Dmrt3*. From this recombination map, *Dmrt1* clearly has much tighter linkage with *Dmrt3* than with *Kank1* (~1 cM vs. 25 cM), suggesting that *Kank1* and *Dmrt1* lie much further apart on the physical map than expected (e.g., as a result of an inversion), or are separated by a strong recombination hotspot.

Sibship analyses confirmed that the *Dmrt1‐1* and *Dmrt3* alleles identified as male specific in Tvedöra were indeed located on nonrecombining Y haplotypes. The most common Y haplotype had fixed allele *174* at *Kank1*,* 294* at *Dmrt1‐1*,* 198* at *Dmrt1‐2*,* 301* at *Dmrt1‐5*, and *281* at *Dmrt3*. Three other closely related Y haplotypes differed at one or two loci (changes to allele *165* or *178* at *Kank1*,* 302* at *Dmrt1‐5*, and/or *276* at *Dmrt3*). These analyses also revealed a highly significant association between inheritance of a male‐specific Y haplotype and offspring phenotypic sex (Table 2), both in metamorphs (*n* = 240, *Dxy* = 0.59, *P *=* *3.8 × 10^−15^) and in froglets (*n* = 83, *Dxy* = 0.56; *P *=* *2.2 × 10^−8^). Among the six families analyzed, five turned out to possess a Y haplotype, which correlated significantly with offspring maleness score, although with some variation among families (*n* = 47–60 each, *Dxy* ranging 0.12–0.59). The only family lacking a Y haplotype (ST_1_) displayed an extremely female‐biased sex ratio (50 daughters vs. one son).

**Figure 4:**
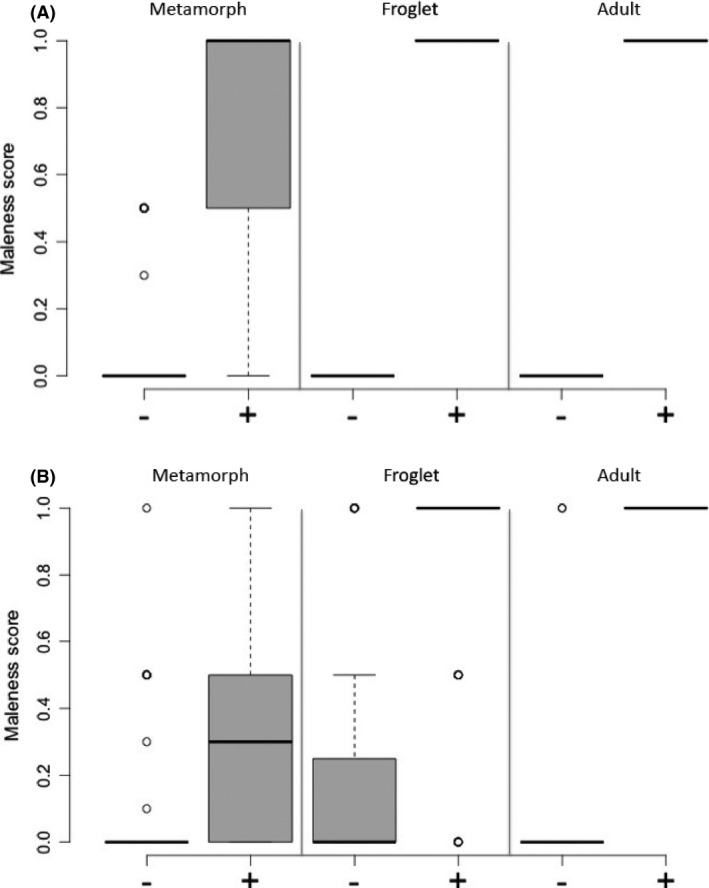
Boxplots of maleness scores for individuals with (+) or without (−) the local Y‐specific *Dmrt1‐1* alleles in metamorphs, froglets, and adults from Ammarnäs (A) and Tvedöra (B).

In both populations, the male specificity of local Y haplotypes, as measured by *Dxy*, increased from the juvenile to the adult stages: In Ammarnäs, sex association was imperfect among metamorphs (*Dxy* = 0.71; Fig. 4A), mostly due to some offspring with undifferentiated gonads and two XY females, but perfect in both froglets and adults (*Dxy* = 1.0). In Tvedöra, *Dxy* was below 0.60 in juveniles (Fig. 4B), mostly due to frequent XY individuals with ovaries, but reached 0.82 in adults, where no female had a Y haplotype, while two males lacked it.

## Discussion

The first and main aim of this study was to identify a small sex‐linked region on LG_2_ in a population from the “semidifferentiated race,” in which previous studies had failed to find any XY differentiation despite strong evidence for a role of this linkage group in sex determination. This aim was entirely fulfilled: our genotyping of adult males and females from Tvedöra uncovered a small nonrecombining segment on LG_2_ that displays significant XY differentiation (Fig. 3). Male‐specific alleles were identified at *Dmrt1‐1* and *Dmrt3* but not at *Dmrt1‐2* and *Dmrt1‐5*, which lie in‐between (and thus necessarily also belong to the nonrecombining segment) but had fixed alleles on the Y haplotype that also segregate on the X chromosomes. Sex association was further confirmed by sibship analyses, which showed a strong association between offspring phenotypic sex and inheritance of the local Y haplotype (Fig. 4). This result constitutes an important step toward the identification of the sex locus, given that all other LG_2_ markers investigated so far showed no differentiation.

This differential segment is much shorter in Tvedöra than in Ammarnäs, with an estimated length on the female recombination map ranging between 0.8 cM (distance between *Dmrt1‐1* and *Dmrt3*) and 23 cM (distance between *Bfg191* and *Bfg093*), as compared to a minimal length of 143 cM in Ammarnäs (distance between *Bfg131* and *Bfg147*). It is also less differentiated, with an *F*_ST_ of 0.061 as compared to 0.230 in Ammarnäs for this specific region (averages over the *Dmrt* markers). The Tvedöra and Ammarnäs Y haplotypes differ in fact markedly, bearing distinct alleles at each of the four *Dmrt* markers (as opposed to the X‐linked alleles that are largely shared).

This smaller and less differentiated SDR associates with weaker masculinizing effects. The five Tvedöra families with a Y haplotype displayed lower *Dxy* values than Ammarnäs families, mostly due to a high number of XY individuals presenting ovaries at the metamorph and froglet stages. Interestingly, these discrepancies between phenotypic and genotypic sex decreased between the juvenile and adult stages, suggesting that sex differentiation can be delayed beyond the froglet stage in the “semidifferentiated race.” Occasional XY females that reach reproductive age might actually account for the overall absence of XY differentiation in Tvedöra, as recombination patterns in frogs seem to depend on phenotypic rather than genotypic sex (the fountain‐of‐youth hypothesis; Perrin 2009; Matsuba et al. 2010). Reciprocally, X‐specific haplotypes in Tvedöra seemingly have weaker feminizing effects, as shown by the occurrence of XX males. The progeny of one of the two males (of 11) that lacked a Y haplotype could be analyzed and revealed an extreme female bias (50 daughters for one son), further supporting an XX paternal genotype. This result confirms that sex reversals account for some of the variance in sex ratios among families and provides further support for a sex‐determining role of the Y haplotypes identified here.

It is obviously of interest that the small nonrecombining segment in Tvedöra encompasses *Dmrt1*, a gene from the sex‐determining cascade that plays a key role in sex determination and sexual dimorphism throughout all metazoans. Whether this gene is directly involved in the patterns documented here (i.e., is the sex locus), or only turned out by chance to be trapped in the nonrecombining segment, is an open question. The classical paradigm of sex‐chromosome evolution predicts absence of Y polymorphism in the SDR (as a result of complete arrest of XY recombination and ensuing strong genetic drift and Hill‐Robertson interferences), which does not fit with the *Dmrt1* polymorphism documented here. However, this classical paradigm was specifically developed to account for the highly differentiated sex chromosomes documented in lineages with purely GSD such as mammals, birds, and *Drosophila*; it has little relevance for systems with homomorphic sex chromosomes such as found in many fish, amphibians, and nonavian reptiles, where nongenetic effects may also contribute to sex determination. Sex reversals and occasional XY recombination are expected to refuel the genetic variance at the SDR. In the specific case of *R. temporaria*, furthermore, the patterns of sex determination and gonadal differentiation are known to be polymorphic both within and among populations (Witschi 1929, 1930; Rodrigues et al. 2013, 2014, 2015); sex determination varies from entirely genetic in some families to entirely nongenetic in others (e.g., Brelsford et al. 2016c). Hence, some polymorphism is indeed expected at the SDR.

This issue will clearly not be settled with data in hand, but our results do suggest further investigations that might help to clarify this point. Extension of analyses in Tvedöra to genomic regions between *Dmrt1* and *Kank1* (which does not seem to belong to the SDR), and downstream of *Dmrt3* (which is apparently involved), might help evaluate more precisely the extent of the SDR and possibly identify alternative candidate genes. Similar analyses in Ammarnäs would not be informative, given that most of the sex chromosome belongs to the nonrecombining SDR. Although the strongly masculinizing/feminizing effects of sex‐specific haplotypes in Ammarnäs might possibly stem from the distinct *Dmrt1* alleles segregating in this population, linkage with other genes from the sex‐determining pathway located on the same chromosome (such as *Amh*) is expected to contribute as well.

Investigations of polymorphisms in this genomic region should also be extended to a broader geographic scale. The “differentiated sex race” occurs in both alpine and boreal climates (Witschi 1930). It would be worth checking whether the same *Dmrt1* Y haplotypes as in Ammarnäs are found in Alpine populations, or whether different Y haplotypes independently evolved in these distinct geographic areas. Similarly, populations from the “undifferentiated sex race,” spread in milder climates (from southern England, Netherlands, and central Germany down to the Jura mountains; Witschi 1930) should be investigated for the same markers. If sex determination in the undifferentiated sex race is purely nongenetic, as hypothesized by Rodrigues et al. (2015), then we predict a complete absence of sex differentiation in the genomic region surrounding *Dmrt1*. On a broader scale, the question arises whether the “sex races” described in other species of Ranidae (e.g., Pflüger 1881; Swingle 1926; Hsü and Liang 1970; Gramapurohit et al. 2000) also differ in the size and differentiation of nonrecombining segments on their sex chromosomes.

It is worth noting that the chromosome pair under focus, corresponding to *X. tropicalis* scaffold 1, has been independently co‐opted for sex determination in different lineages of amphibians, including species of Bufonidae, Hylidae and Ranidae (e.g., Sumida and Nishioka 2000; Miura 2007; Brelsford et al. 2013; Dufresnes et al. 2015). Recent investigations on four European species of tree frogs from the *Hyla arborea* group have furthermore shown these species to share a small SDR that also contains *Dmrt1* (Brelsford et al. 2016b). Hence, our results substantiate the view that such recurrent convergences of sex determination toward a limited set of chromosome pairs might result from the co‐option of small genomic regions that harbor key genes from the sex‐determination pathway (Graves and Peichel 2010; O'Meally et al. 2012; Brelsford et al. 2013).

## Conflict of Interest

None declared.

## Data Accessibility

Genotyping data of all *Dmrt1* markers are deposited in Dryad database, doi:10.5061/dryad.kp296.

## Supporting information


**Appendix S1.**
**Text S1.** Seven scaffolds from the draft genome of *Rana temporaria*, containing, respectively, the five *Dmrt1* exons, *Kank1* intron 1, and *Dmrt3* intron 1.
**Text S2.** Transcript sequences of *R. temporaria Dmrt1* in five froglets.
**Text S3.** Concatenated sequences of three *Dmrt1* polymorphic sites for 26 individuals from Ammarnäs and Tvedöra.

**Appendix S2**
**Table S1.** Primer pairs and PCR conditions for amplifying *Dmrt1* transcript and individual exons.
**Table S2.** Primers pairs and PCR conditions for genotyping.
**Table S3.** Between‐sex *F*_ST_ values in Ammarnäs and Tvedöra.

## References

[ece32209-bib-0001] Alho, J. S. , C. Matsuba , and J. Merilä . 2010 Sex reversal and primary sex ratios in the common frog (*Rana temporaria*). Mol. Ecol. 19:1763–1773. 20345673 10.1111/j.1365-294X.2010.04607.x

[ece32209-bib-0002] Beukeboom, L. W. , and N. Perrin . 2014 The evolution of sex determination. Oxford Univ. Press, Oxford, UK.

[ece32209-bib-0003] Brelsford, A. , M. Stöck , C. Betto‐Colliard , S. Dubey , C. Dufresnes , H. Jourdan‐Pineau , et al. 2013 Homologous sex chromosomes in three deeply divergent anuran species. Evolution 67:2434–2440. 23888863 10.1111/evo.12151

[ece32209-bib-0004] Brelsford, A. , C. Dufresnes , and N. Perrin . 2016a High‐density sex‐specific linkage maps of a European tree frog (*Hyla arborea*) identify the sex chromosome without information on offspring sex. Heredity 116:177–181. 26374238 10.1038/hdy.2015.83 PMC4806884

[ece32209-bib-0005] Brelsford, A. , C. Dufresnes , and N. Perrin . 2016b Trans‐species variation in *Dmrt1* is associated with sex determination in four European tree‐frog species. Evolution 70:840–847. 26920488 10.1111/evo.12891

[ece32209-bib-0006] Brelsford, A. , N. Rodrigues , and N. Perrin . 2016c High‐density linkage maps fail to detect any genetic component to sex determination in a *Rana temporaria* family. J. Evol. Biol. 29:220–225. 26404414 10.1111/jeb.12747

[ece32209-bib-0007] Cano, J. M. , M. H. Li , A. Laurila , J. Vilkki , and J. Merilä . 2011 First‐generation linkage map for the common frog *Rana temporaria* reveals a sex linkage group. Heredity 107:530–536. 21587305 10.1038/hdy.2011.39 PMC3242625

[ece32209-bib-0008] Devlin, R. H. , and Y. Nagahama . 2002 Sex determination and sex differentiation in fish: an overview of genetic, physiological, and environmental influences. Aquaculture 208:191–363.

[ece32209-bib-0009] Dufresnes, C. , A. Borzée , A. Horn , M. Stöck , M. Ostini , R. Sermier , et al. 2015 Sex‐chromosome homomorphy in Palearctic tree frogs results from both turnovers and X‐Y recombination. Mol. Biol. Evol. 32:2328–2337. 25957317 10.1093/molbev/msv113

[ece32209-bib-0010] Eggert, C. 2004 Sex determination: the amphibian models. Reprod. Nutr. Dev. 44:539–549. 15762298 10.1051/rnd:2004062

[ece32209-bib-0011] Evans, B. J. , R. A. Pyron , and J. J. Wiens . 2012 Polyploidization and sex chromosome evolution in amphibians Pp. 385–410 *in* Soltis P. S. and Soltis D. E., eds. Polyploidy and genome evolution. Springer, Heidelberg.

[ece32209-bib-0012] Gosner, K. L. 1960 A simplified table for staging anuran embryos and larvae with notes on identification. Herpetologica 16:183–190.

[ece32209-bib-0013] Goudet, J. 1995 FSTAT (version 1.2): a computer program to calculate F‐statistics. J. Hered. 86:485–486.

[ece32209-bib-0014] Gramapurohit, N. P. , B. A. Shanbhag , and S. K. Saidapur . 2000 Pattern of gonadal sex differentiation, development, and onset of steroidogenesis in the frog, *Rana curtipes* . Gen. Comp. Endocrinol. 119:256–264. 11017773 10.1006/gcen.2000.7513

[ece32209-bib-0015] Graves, J. A. M. , and C. L. Peichel . 2010 Are homologies in vertebrate sex determination due to shared ancestry or to limited options? Genome Biol. 11:205–217. 20441602 10.1186/gb-2010-11-4-205 PMC2884537

[ece32209-bib-0016] Green, P. , K. Falls , and S. Crook . 1990 Documentation for CRIMAP, version 2.4. St. Louis, MO: Washington Univ. School of Medicine.

[ece32209-bib-0017] Grossen, C. , S. Neuenschwander , and N. Perrin . 2011 Temperature‐dependent turnovers in sex‐determination mechanisms: a quantitative model. Evolution 65:64–78. 20722730 10.1111/j.1558-5646.2010.01098.x

[ece32209-bib-0018] Guerrero, R. , M. Kirkpatrick , and N. Perrin . 2012 Cryptic recombination in the ever‐young sex chromosomes of Hylid frogs. J. Evol. Biol. 25:1947–1954. 22901240 10.1111/j.1420-9101.2012.02591.x

[ece32209-bib-0019] Haczkiewicz, K. , and M. Ogielska . 2013 Gonadal differentiation in frogs: how testes become shorter than ovaries. Zoolog. Sci. 30:125–134. 23387847 10.2108/zsj.30.125

[ece32209-bib-0020] Hillis, D. M. , and D. M. Green . 1990 Evolutionary changes of heterogametic sex in the phylogenetic history of amphibians. J. Evol. Biol. 3:49–64.

[ece32209-bib-0021] Hsü, C. Y. , and H. M. Liang . 1970 Sex races of *Rana catesbeiana* in Taiwan. Herpetologica 26:214–221.

[ece32209-bib-0022] Matsuba, C. , I. Miura , and J. Merilä . 2008 Disentangling genetic vs. environmental causes of sex determination in the common frog, *Rana temporaria* . BMC Genet. 9:3. 18182101 10.1186/1471-2156-9-3 PMC2265737

[ece32209-bib-0023] Matsuba, C. , J. S. Alho , and J. Merilä . 2010 Recombination rate between sex chromosomes depends on phenotypic sex in the common frog. Evolution 64:3634–3637. 20624177 10.1111/j.1558-5646.2010.01076.x

[ece32209-bib-0024] Matsuda, M. , Y. Nagahama , A. Shinomiya , T. Sato , C. Matsuda , T. Kobayashi , et al. 2002 *DMY* is a Y‐specific DM‐domain gene required for male development in the medaka fish. Nature 417:559–563. 12037570 10.1038/nature751

[ece32209-bib-0025] Miura, I. 2007 An evolutionary witness: the frog *Rana rugosa* underwent change of heterogametic sex from XY male to ZW female. Sex. Dev. 1:323–331. 18391544 10.1159/000111764

[ece32209-bib-0026] Nanda, I. , M. Kondo , U. Hornung , S. Asakawa , C. Winkler , A. Shimizu , et al. 2002 A duplicated copy of *DMRT1* in the sex‐determining region of the Y chromosome of the medaka, *Oryzias latipes* . Proc. Natl Acad. Sci. USA 99:11778–11783. 12193652 10.1073/pnas.182314699 PMC129345

[ece32209-bib-0027] Ogielska, M. , and A. Kotusz . 2004 Pattern and rate of ovary differentiation with reference to somatic development in anuran amphibians. J. Morphol. 259:41–54. 14666524 10.1002/jmor.10162

[ece32209-bib-0028] O'Meally, D. , T. Ezaz , A. Georges , S. D. Sarre , and J. A. M. Graves . 2012 Are some chromosomes particularly good at sex? Insights from amniotes Chromosome Res. 20:7–19. 22218935 10.1007/s10577-011-9266-8

[ece32209-bib-0029] Perrin, N. 2009 Sex reversal: a fountain of youth for sex chromosomes? Evolution 63:3043–3049. 19744117 10.1111/j.1558-5646.2009.00837.x

[ece32209-bib-0030] Pflüger, E. 1881 Über das geschlechtsbestimmenden Ursachen und die Geschlechtsverhältnisse der Frösche. Arch. F. Gesamte Phys. 29:13–40.

[ece32209-bib-0031] R Core Team . 2014 R: a language and environment for statistical computing. R Foundation for Statistical Computing, Vienna, Austria http://www.R-project.org/.

[ece32209-bib-0032] Rodrigues, N. , C. Betto‐Colliard , H. Jourdan‐Pineau , and N. Perrin . 2013 Within‐population polymorphism of sex‐determination systems in the common frog (*Rana temporaria*). J. Evol. Biol. 26:1569–1577. 23711162 10.1111/jeb.12163

[ece32209-bib-0033] Rodrigues, N. , J. Merilä , C. Patrelle , and N. Perrin . 2014 Geographic variation in sex‐chromosome differentiation in the common frog (*Rana temporaria*). Mol. Ecol. 23:3409–3418. 24935195 10.1111/mec.12829

[ece32209-bib-0034] Rodrigues, N. , Y. Vuille , J. Loman , and N. Perrin . 2015 Sex‐chromosome differentiation and ‘sex races’ in the common frog (*Rana temporaria*). Proc. R. Soc. B. 282:20142726. 10.1098/rspb.2014.2726 PMC4426610 25833852

[ece32209-bib-0035] Rodrigues, N. , Y. Yuille , A. Bresford , and N. Perrin . 2016 The genetic contribution to sex determination and number of sex chromosomes vary among populations of common frogs (*Rana temporaria*). Heredity. doi:10.1038/hdy.2016.22. 10.1038/hdy.2016.22 PMC4901354 27071845

[ece32209-bib-0036] Schartl, M. 2004 Sex chromosome evolution in non‐ mammalian vertebrates. Curr. Opin. Genet. Dev. 14:634–641. 15531158 10.1016/j.gde.2004.09.005

[ece32209-bib-0037] Schmid, M. , and C. Steinlein . 2001 Sex chromosomes, sex‐linked genes, and sex determination in the vertebrate class Amphibia Pp. 143–176 *in* Scherer G., Schmid M., eds. Genes and mechanisms in vertebrate sex determination. Birkhäuser Verlag, Basel. 10.1007/978-3-0348-7781-7_8 11301597

[ece32209-bib-0038] Schmid, M. , I. Nanda , C. Steinlein , K. Kausch , and T. Haaf . 1991 Sex‐determining mechanisms and sex chromosomes in amphibian Pp. 393–430 *in* Green D. M. and Sessions S. K., eds. Amphibian cytogenetics and evolution. Academic Press, San Diego.

[ece32209-bib-0039] Schmid, M. , C. Steinlein , J. P. Bogart , W. Feichtinger , P. León , E. La Marca , et al. 2010 The chromosomes of terraranan frogs. Insights into vertebrate cytogenetics. Cytogenet. Genome Res. 129:1–4. 21063086 10.1159/000301339

[ece32209-bib-0040] Smith, C. A. , K. N. Roeskler , T. Ohnesorg , D. M. Cummins , P. G. Farlie , T. J. Doran , et al. 2009 The avian Z‐linked gene *DMRT1* is required for male sex determination in the chicken. Nature 461:267–271. 19710650 10.1038/nature08298

[ece32209-bib-0041] Somers, R. H. 1962 A new asymmetric measure of association for ordinal variables. Am. Sociol. Rev. 27:799–811.

[ece32209-bib-0042] Stöck, M. , A. Horn , C. Grossen , D. Lindtke , R. Sermier , C. Betto‐Colliard , et al. 2011 Ever‐young sex chromosomes in European tree frogs. PLoS Biol. 9:e1001062. 21629756 10.1371/journal.pbio.1001062 PMC3100596

[ece32209-bib-0043] Stöck, M. , R. Savary , C. Betto‐Colliard , S. Biollay , H. Jourdan‐Pineau , and N. Perrin . 2013 Low rates of X‐Y recombination, not turnovers, account for homomorphic sex chromosomes in several diploid species of Palearctic green toads (*Bufo viridis* subgroup). J. Evol. Biol. 26:674–682. 23316809 10.1111/jeb.12086

[ece32209-bib-0044] Sumida, M. , and M. Nishioka . 2000 Sex‐linked genes and linkage maps in amphibians. Comp. Biochem. Physiol. B Biochem. Mol. Biol. 126:257–270. 10874173 10.1016/s0305-0491(00)00204-2

[ece32209-bib-0045] Swingle, W. W. 1926 The germ cells of anurans. II. An embryological study of sex differentiation in *Rana catesbeiana* . J. Morphol. 41:441–546.

[ece32209-bib-0046] Volff, J. N. , I. Nanda , B. Schmid , and M. Schartl . 2007 Governing sex determination in fish: regulatory putches and ephemeral dictators. Sex. Dev. 1:85–99. 18391519 10.1159/000100030

[ece32209-bib-0047] Voorrips, R. E. 2002 MapChart: software for graphical presentation of linkage maps and QTLs. J. Hered. 93:77–78. 12011185 10.1093/jhered/93.1.77

[ece32209-bib-0048] Witschi, E. 1929 Studies on sex differentiation and sex determination in amphibians. III. Rudimentary hermaphroditism and Y chromosome in *Rana temporaria* . J. Exp. Zool. 54:157–223.

[ece32209-bib-0049] Witschi, E. 1930 Studies on sex differentiation and sex determination in amphibians. IV. The geographical distribution of the sex races of the European grass frog (*Rana temporaria*, L.). J. Exp. Zool. 56:149–165.

[ece32209-bib-0050] Yoshimoto, S. , E. Okada , H. Umemoto , K. Tamura , Y. Uno , C. Nishida‐Umehara , et al. 2008 A W‐linked DM‐domain gene, *DM‐W*, participates in primary ovary development in *Xenopus laevis* . Proc. Natl Acad. Sci. USA 105:2469–2474. 18268317 10.1073/pnas.0712244105 PMC2268160

