# Supplementary material for: Dmrt1 polymorphism covaries with sex‐determination patterns in Rana temporaria
Source: Ecol Evol. 2016 May 30;6(15):5107–17. doi: 10.1002/ece3.2209 (PMC4891206; doi:10.1002/ece3.2209)
Supplement: Supplementary file 2 — Appendix S2 Table S1. Primer pairs and PCR conditions for amplifying Dmrt1 transcript and individual exons. Table S2. Primers pairs and PCR conditions for genotyping. Table S3. Between‐sex F ST values in Ammarnäs and Tvedöra. [file ECE3-6-5107-s002.doc]

| Table 1) Primer pairs and polymerase chain reaction (PCR) conditions for amplifying *Dmrt1* transcript and individual exons. | | | |
| --- | --- | --- | --- |
| Locus_name | Forward_primer | Reverse_primer | PCR conditions |
| ***Dmrt1*_exon1** | TGTCTCAGAGCAAGAATCAC | TTTACAGTGCTAACCACACCA | 3min at 95°C, followed by 35 cycles of 30s at 95°C, 30s at 54°C and 30s at 72°C, finally elongation of 5min at 72°C |
| ***Dmrt1*_exon2** | TTTAGGAAATGTAGGAGGAAACGG | GGAATTTCTGCTCTGCTCCCT | 3min at 95°C, followed by 35 cycles of 30s at 95°C, 30s at 52°C and 30s at 72°C, finally elongation of 5min at 72°C |
| ***Dmrt1*_exon3** | CGTCTATGTGAATGTAACCGT | GTTAAGAACAGCACAGGAGG | 3min at 95°C, followed by 35 cycles of 30s at 95°C, 30s at 56°C and 30s at 72°C, finally elongation of 5min at 72°C |
| ***Dmrt1*_exon4** | ACGCTGCTTCAGATTCTACAC | AAACTTACCACTTGACTTGACCTC | 3min at 95°C, followed by 35 cycles of 30s at 95°C, 30s at 53°C and 30s at 72°C, finally elongation of 5min at 72°C |
| ***Dmrt1*_exon5** | GGATTCTGGATTGGTTTCTTTGTC | CTAACTTGTATGGCTAGACCGT | 3min at 95°C, followed by 35 cycles of 30s at 95°C, 30s at 55°C and 30s at 72°C, finally elongation of 5min at 72°C |
| ***Dmrt1*_exon1-5** | GAGCCATTAAGCAAGCCTCG | CTAACTTGTATGGCTAGACCGT | 3min at 95°C, followed by 35 cycles of 30s at 95°C, 30s at 56°C and 30s at 72°C, finally elongation of 5min at 72°C |
| ***Dmrt1*_exon2-3** | CAACAAGCCCAAGAAGAGGA | GCCATTGGTTTCCAGCTTGTC | 3min at 95°C, followed by 35 cycles of 30s at 95°C, 30s at 56°C and 30s at 72°C, finally elongation of 5min at 72°C |
| ***Dmrt1*_exon2-4** | CAACAAGCCCAAGAAGAGGA | CCTCAAATGTAAAGAATGGTGG | 3min at 95°C, followed by 35 cycles of 30s at 95°C, 30s at 56°C and 30s at 72°C, finally elongation of 5min at 72°C |
|  |  |  |  |
| Note: *Dmrt1* exon1 primer pair amplifies the sequence of exon1 and both flanking regions (< 200bp each direction). | | | |
| *Dmrt1*_exon2 primer pair amplifies the sequence of exon2 and 5’ part of flanking region (< 200bp each direction). | | | |
| *Dmrt1* _ exon3 primer pair amplifies the sequence of exon3 and both flanking regions (< 200bp each direction). | | | |
| *Dmrt1* _ exon4 primer pair amplifies the sequence of exon4 and both flanking regions (< 200bp each direction). | | | |
| *Dmrt1*_exon5 primer pair amplifies the sequence of exon5 and 3’ part of flanking region (< 200bp each direction), including the beginning of 3'UTR region. | | | |
| *Dmrt1*_exon1-5 primer pair amplifies the sequence from the beginning of exon1 until the end of exon5. | | | |
| *Dmrt1*_exon2-3 primer pair amplifies the sequence from the beginning of exon2 until the end of exon3. | | | |
| *Dmrt1*_exon2-4 primer pair amplifies the sequence from the beginning of exon2 until the end of exon4. | | | |

Table 2). Primers and PCR conditions for genotyping.

| Locus | Forward primer | Reverse primer | PCR conditions |
| --- | --- | --- | --- |
| *Kank1* | TTGTGAAAATGGCAATGAATG | AGTGGCAAAGGAACCTGTAAA | 15min at 95°C, followed by 35 cycles of 30s at 94°C, 1.5min at 55°C and elongation at 72°C for 1min, a final elongation of 30min at 60°C |
| *Dmrt1*_1 | GGCTATTCGTCGCTACTAAAGG | AAGATCTCGCATTTACAAGAG |
| *Dmrt1*_2 | GGTCATTCCTTGTCCTAATTATCAGT | GCCATAATAAGCTGGGTCAACC |
| *Dmrt1*_5 | GGATTCTGGATTGGTTTCTTTGTC | GTGGCTTATGCCATGCAGAT |
| *Dmrt3* | GGCTGGGAACACAGATATGG | CTTTGTGGCTTCCCTGCTTTA |

Table 3). Between-sex F_ST_ values in Ammarnäs and Tvedöra. Markers are listed according to their position on the female recombination map (Fig. 2). Indicated in bold are markers developed for this study, as well as p-values significant after correction for multiple testing.

| Ammarnäs | | | | Tvedöra | | |
| --- | --- | --- | --- | --- | --- | --- |
| Markers | F_ST_ | P  (uncorrected) | P (with Benjamini-Hochberg correction) | F_ST_ | P  (uncorrected) | P (with Benjamini-Hochberg correction) |
| *Bfg092* | 0.039 | 0.1057 | 0.1119 | -0.029 | 0.9819 | 0.9819 |
| *BFG131* | 0.089 | 0.0016 | **0.0021** | -0.037 | 0.9818 | 0.9819 |
| *BFG172* | 0.078 | 0.0003 | **0.0004** | 0.014 | 0.2127 | 0.5504 |
| *BFG053* | 0.29 | 0.0001 | **0.0002** | 0.042 | 0.0267 | 0.1602 |
| ***Kank1*** | 0.514 | 0.0001 | **0.0002** | -0.033 | 0.2944 | 0.5504 |
| *BFG191* | 0.062 | 0.0002 | **0.0003** | 0.01 | 0.2273 | 0.5504 |
| ***Dmrt1-1*** | 0.239 | 0.0001 | **0.0002** | 0.167 | 0.0002 | **0.0036** |
| ***Dmrt1-2*** | 0.268 | 0.0001 | **0.0002** | 0.007 | 0.0922 | 0.4149 |
| ***Dmrt1-5*** | 0.269 | 0.0001 | **0.0002** | -0.014 | 0.6798 | 0.9041 |
| ***Dmrt3*** | 0.142 | 0.0001 | **0.0002** | 0.084 | 0.0097 | 0.0873 |
| *BFG093* | 0.167 | 0.0002 | **0.0003** | -0.018 | 0.9251 | 0.9819 |
| *RtuB* | 0.067 | 0.0397 | **0.0447** | 0.016 | 0.2157 | 0.5504 |
| *BFG266* | 0.058 | 0.0001 | **0.0002** | -0.008 | 0.3981 | 0.6514 |
| *RFG021* | 0.018 | 0.1438 | 0.1438 | 0.015 | 0.3058 | 0.5504 |
| *RTSB03* | 0.163 | 0.0001 | **0.0002** | -0.015 | 0.5377 | 0.8066 |
| *Rtemp5* | 0.289 | 0.0001 | **0.0002** | -0.028 | 0.8398 | 0.9819 |
| *BFG072* | 0.105 | 0.0168 | **0.0202** | -0.019 | 0.7032 | 0.9041 |
| *Bfg147*  Overall | 0.036  0.153 | 0.0001  0.0001 | **0.0002** | 0.043  0.011 | 0.2789  0.2398 | 0.5504 |
